# Serological markers and risk factors associated with Hepatitis B virus infection among Federal Capital Territory prison inmates, Nigeria: Should we be concerned?

**DOI:** 10.1371/journal.pone.0248045

**Published:** 2021-03-11

**Authors:** Chioma Cindy Dan-Nwafor, Ikeola Adeoye, Kehinde Aderemi, Martins Onuoha, Elizabeth Adedire, Adebobola Bashorun, Damaris Osunkwo, Saheed Gidado, Muhammad Balogun, Suleiman Idris, Ibrahim Ade-Yusuf, Ekpedeme Udom, Patrick Nguku

**Affiliations:** 1 Nigeria Field Epidemiology and Laboratory Training Programme, Decatur, Georgia, United States of America; 2 Department of Epidemiology and Medical Statistics, University of Ibadan, Ibadan, Nigeria; 3 Department of Medical Microbiology and Parasitology, College of Medicine University of Ibadan, Ibadan, Nigeria; 4 Nigerian Prisons Service Headquarters, Abuja, Nigeria; 5 Department of Community Medicine, Ahmadu Bello University, Zaria, Nigeria; University of Cincinnati College of Medicine, UNITED STATES

## Abstract

**Introduction:**

Hepatitis B virus (HBV) infection is hyper-endemic in Nigeria. Prisons are high-risk environments for the spread of infectious diseases. Worldwide, seroprevalence of HBV infection is substantially higher among individuals in correctional facilities when compared to general population. We determined the seroprevalence and risk factors associated with HBV infection among Kuje prison inmates, Nigeria.

**Material and methods:**

We conducted a prison facility based cross-sectional study. Interviewer administered questionnaires were used to obtain information on participants socio-demographic characteristics, HBV risk factors, previous HBV test and vaccination history. Blood samples collected from participants were analysed for HBsAg, HBsAb, HBcAb, HBeAg and HBeAb markers using rapid lateral chromatographic immunoassay kit. Univariate, bivariate, and multivariate analysis were performed.

**Results:**

A total of 271 inmates (63 convicts and 208 awaiting trial inmates) were recruited into the study as participants. The mean age of the participants was 32.7 SD±9 years. HBV seroprevalence (HBsAg) of 13.7% (95% CI: 9.8–18.3) was found. 55.4% (95% CI: 49.2–61.4) of inmates were susceptible to HBV infection, 20.7% (95%CI; 16.0–26.0) had past HBV infection while 10.3% (95% CI: 7.0–14.6) had acquired natural or artificial HBV immunity. Factors found to be associated with current HBV infection (HBsAg) include age-group ≤25years (aOR = 8.0,95% CI: 2.9–22.3), being ever married (aOR = 4.2, 95% CI: 1.7–10.4) and history of alcohol consumption (aOR = 3.4, 95% CI: 1.3–8.4).

**Conclusion:**

This study reveals a high seroprevalence of HBV infection among Kuje Prison inmates, hence the need to introduce prison-focused health intervention initiatives such as HBV screening, vaccination and care to reduce the transmission of HBV infection among inmates and ultimately the general population.

## Introduction

Prisons form part of the criminal justice system. It is estimated that over nine million people are in penal institutions worldwide, with 73,995 incarcerated in Nigeria as at July 2019 [1, 2]. In comparison with the general population, individuals incarcerated in correctional institutions bear a incommensurably greater burden of infectious diseases, including Hepatitis B virus (HBV) infection [3, 4].

HBV is a major global health problem and is an important cause of death worldwide [5, 6]. Complications of chronic HBV infection such as chronic hepatitis, cirrhosis, and hepatocellular carcinoma account for 500,000 to 1.2 million deaths yearly [7, 8]. The most common effect of HBV infection is the expression of various serological markers of varying epidemiological and clinical significance. These HBV viral markers serve as serologic evidence of HBV acute, chronic infection and immunity infectivity status. Combination of these HBV markers form HBV serological patterns with diverse health implications and interventions among inmates [9].

In Africa, seroprevalence of HBV infection ranges from 8–20% accounting for second largest number of chronic HBV carriers after Asia, with sub-Saharan Africa bearing the highest burden [10]. Nigeria remains hyper-endemic for HBV infection, despite the existence of a safe and effective vaccine introduced in Nigeria in 2004 as part of the National Program on Immunization (NPI), to be given at 6, 10, and 14 weeks of age at no cost [11]. Likewise, HBV sero-prevalence of 12.2% was documented in the first Hepatitis B national survey among asymptomatic Nigerians [12]. Due to the increasing seroprevalence of HBV infection in the last decade, WHO designates every July 28 as World Hepatitis Day to increase awareness and encourage HBV infection prevention, diagnosis and care.

Some recognised factors that aid the transmission of HBV among prison inmates include common use of hypodermic needles for execution of tattoos, needle sharing-injection drug use (IDU) and unsafe sexual activity with multiple partners, in addition to inadequate prison health-care services [1, 5, 13, 14]. Although these risky behaviours are strictly not tolerated by the authorities at the correctional facilities, some of these behaviours still continue. There are no prison records to quantify occurrences, thus the need to assess and quantify HBV associated risk factors in the prison [4, 15].

Congestion of prison owing to intake of inmates above the designated official capacity and unhygienic prison conditions are prevalent in Nigerian prisons. As reported by Amnesty International in 2012, about 80% of inmate in more than 240 prison facilities in Nigeria are awaiting trial inmates. This creates overcrowding which aids the spread of infectious disease including HBV [16].

Although some inmates had acquired HBV infection prior to incarceration, there are also growing evidences through prospective cohort studies of ongoing HBV infection transmission among inmates [4, 17, 18]. Moreover, there are no specific health interventions regarding prevention and transmission of HBV infection in Nigerian prisons. Lack of HBV screening and vaccination of inmates has led to asymptomatic carriers fuelling the infection transmission chain through high risk behaviours and practices [19].

Despite the tremendous public health importance of HBV infection there exist sparse published data on Hepatitis B virus infection among prison inmates in Nigeria and the rest of Africa [1, 20, 21]. Although HBV is 50–100 times more infective than Human Immuno deficiency Virus (HIV), previous studies conducted on these high-risk group in Nigeria have focused more on HIV. Furthermore, studies done on HBV infection were focussed on only HBsAg marker, with inadequate data on other HBV serological markers to ascertain the susceptibility, infectivity and immunity status of inmates [22–24]. Baseline information on HBV prevalence in correctional facilities is imperative for the planning of HBV infection preventive measures such as vaccination programmes. Hence, we determined the serological markers and risk factors associated with Hepatitis B virus infection among Federal Capital Territory (FCT) prison inmates.

## Materials and methods

### Study area and design

A cross-sectional study was carried out between the months of January and August 2016 among prison inmates in FCT Medium-security convict Prison. The prison facility serves offenders in FCT as well as neighbouring States. It is a male only convict prison with an installed capacity for 320 prison inmates but currently caters for about 824 inmates- 652 awaiting trial inmates and 172 Convicts.

The Kuje medium security convict Prison is made up of 4 custodies which accommodates different number of inmates ranging from 2 inmates per cell to 150 inmates’ contingent to the type of crime committed and the custody remanded.

### Sample size determination

A representative sample size was determined after having estimated the required minimum sample size using the formula propounded by Kish and Leslie (1965) at a 95% confidence interval and estimate prevalence of 23.0% Hepatitis B infection among prison inmates in Nasarawa state Nigeria [1, 25].

Sample size estimation for single proportions:

$$n=\frac{Z\alpha^{2}pq}{d^{2}}$$

n = minimum sample size

Zα = the standard normal deviate (1.96 for 95% confidence level)

d = the level of accuracy or precision desire, or sampling error (tolerance error 5%)

p = estimate prevalence of Hepatitis B infection among prison inmates in Nasarawa state Nigeria 23.0% (Adoga et al., 2009)

q = The proportion of the population that does not have the characteristic (i.e. 1- p)

$$n=\frac{1.962^{2}(0.23x0.77)}{0.05^{2}}=272$$

### Finite correction for known population less than 10,000

$$nf=\frac{n}{1+\frac{n}{N}}$$

n = Minimum sample size calculated (272)

N = Total population of Kuje prison (824)

$$nf=\frac{272}{1+\frac{272}{824}}=205$$

### Adjusting for 20% non-response rate

$$NR=\frac{n}{1-NR}$$

$$NR=\frac{205}{1-0.2}=256$$

The minimum sample size for this study was estimated at 256 following finite correction for population less than 10,000 and adjusting for 20% non-response rate. However, 271 inmates who consented were recruited in the study.

### Inclusion and exclusion criteria

Prison inmates (regardless of age) who were less than two months in detention prior to commencement of study data collection were excluded from the study. All inmates who gave written informed consent after indicating willingness to participate were included in the study.

### Sampling technique

**Fig 1:**
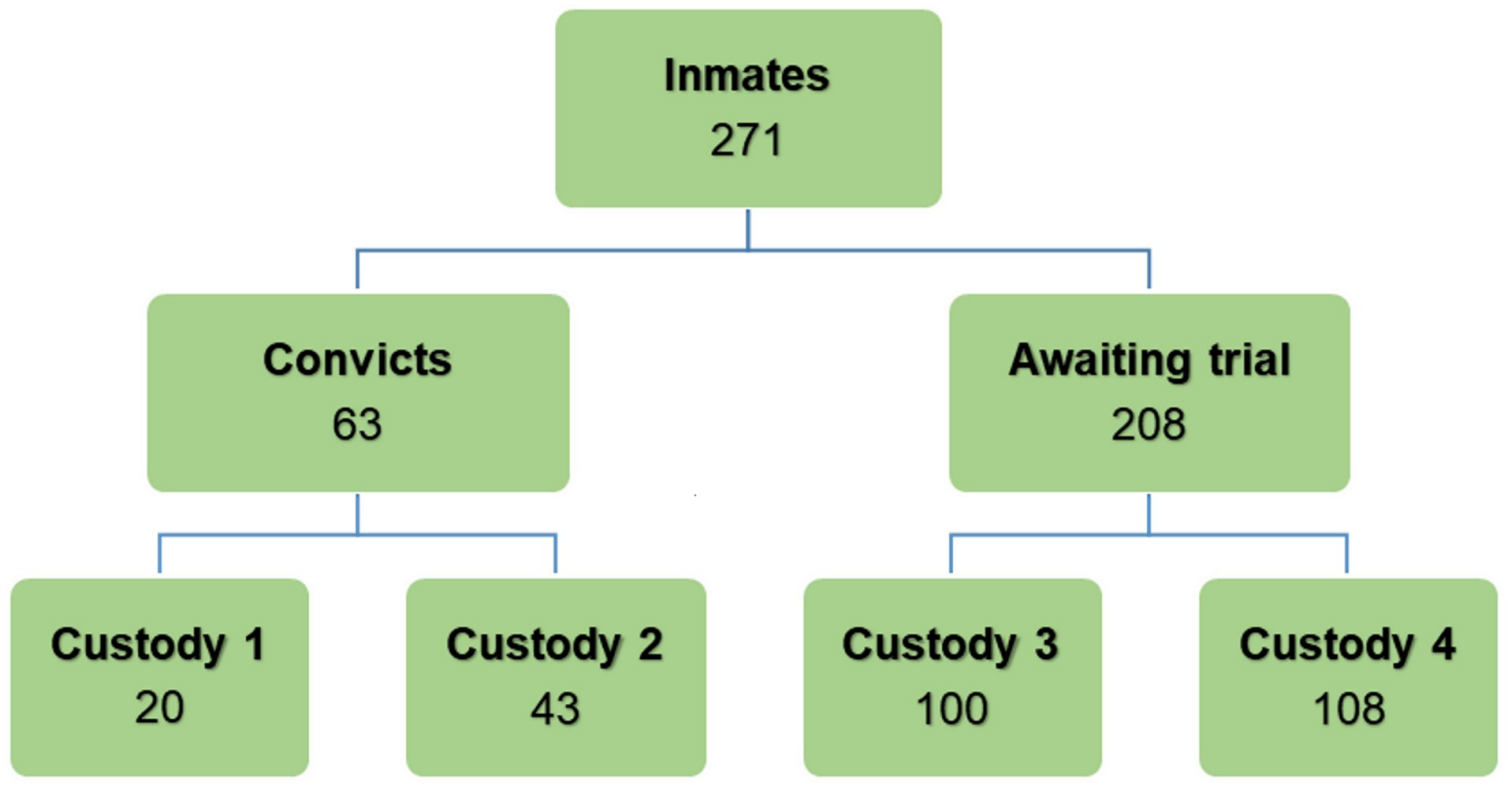
Stratified sampling of study participants.

Stratified sampling technique was used in this study. The inmates were stratified into convict and awaiting trial categories. Probability proportionate to size (PPS) was used to calculate the number of participant in each stratum. Two hundred and seventy-one inmates (63 convicts and 208 awaiting trial inmates) who consented to the study were randomly recruited from all 4 prison custodies to ensure representativeness (Fig 1).

### Ethical considerations

Ethical approval was obtained from FCT Health Research Ethics Committee **FHREC/2015/01/73/23-11-15.**

Permission to conduct the study was obtained from the Nigeria Prison Service Headquarter Office through the Office of the State Controller, FCT Prison Command.

Written consent was obtained from all inmates who participated in the study.

### Data and sample collection

Five millilitres of blood were aseptically collected from each prison inmate by venipuncture of the cubital vein using sterile disposable vacutainer blood collection needles and bottles. Samples were each placed in plain tubes and the sera separated into sample vials and stored at -20°C until analysed. Serum samples were analysed for the presence of HBsAg, HBcAb, HBeAg, HBeAb, and HBsAb, using a rapid lateral flow chromatographic immunoassay kit. The test was carried out and interpreted according to the manufacturer’s instructions. Positive samples were confirmed using Enzyme Linked Immunosorbent assay technique (ELISA).

A pretested structured interviewer administered questionnaire adapted from national Hepatitis survey was used for data collection. The questionnaire included both close and open ended questions and was sectionalised into socio-demographic characteristics, risk factors associated with HBV infection and vaccination history. The laboratory test results for participants were anonymously linked to their questionnaire information through unique identifiers.

### Data analysis

Chi-square was used to test for significant relationship between the dependent and independent variables. P-values were considered significant at < 0.05 and odds ratios were reported with a 95% confidence interval. Multivariate analysis was performed on factors significantly associated with HBV sero-positivity for possible confounding and effect modification. HBV serological marker patterns were interpreted using WHO HBV markers algorithm.

A positive HBsAg test was considered evidence of HBV infection (chronic carrier state or infection) and used to calculate the prevalence, a positive HBcAb test was considered evidence of previous exposure to the HBV, and a positive HBsAb test was considered evidence of being immune to HBV, which when in combination with a positive HBcAb was considered due to natural infection and when alone due to vaccination. Being negative for all markers meant participant was susceptible to HBV infection [26].

## Results

A total of 271 inmates were recruited to participate in the study of which 208 (76.8%) were awaiting trial. The mean age of the participants was 32.7±9 years and 132 (48.7%) were in the age-group 25–34 years. About 107 (46.9%) participants were married, of which 105 (82.7%) were from monogamous homes. Prior to incarceration, the majority of participants (n = 200, (73.8%)) had completed secondary education, while, 224 (82.7%) inmates were self-employed. The majority of participants (n = 180 (66.4%)) were Christians. (Table 1).

**Table 1:** Percentage distribution of study participants by socio-demographic characteristics.

Characteristic	Frequency (n = 271)	Percentage
**Age**		
<25	29	10.7
25–34	132	48.7
35–44	79	29.2
>45	31	11.5
**Marital Status**		
Single	129	47.6
Married	127	46.9
Divorced/Separated/Widowed	15	5.5
**Type of marriage(n = 127)**		
Monogamous	105	82.7
Polygamous	22	17.3
**Highest educational level**		
No formal education	26	9.6
Primary	45	16.6
At least Secondary	200	73.8
**Religion**		
Christianity	180	66.4
Islam	91	33.6
**Occupation before incarceration**		
Public servant	22	8.1
Self employed	133	49.1
Trader	91	33.6
Unemployed	4	1.5
Student	21	7.8

**Fig 2:**
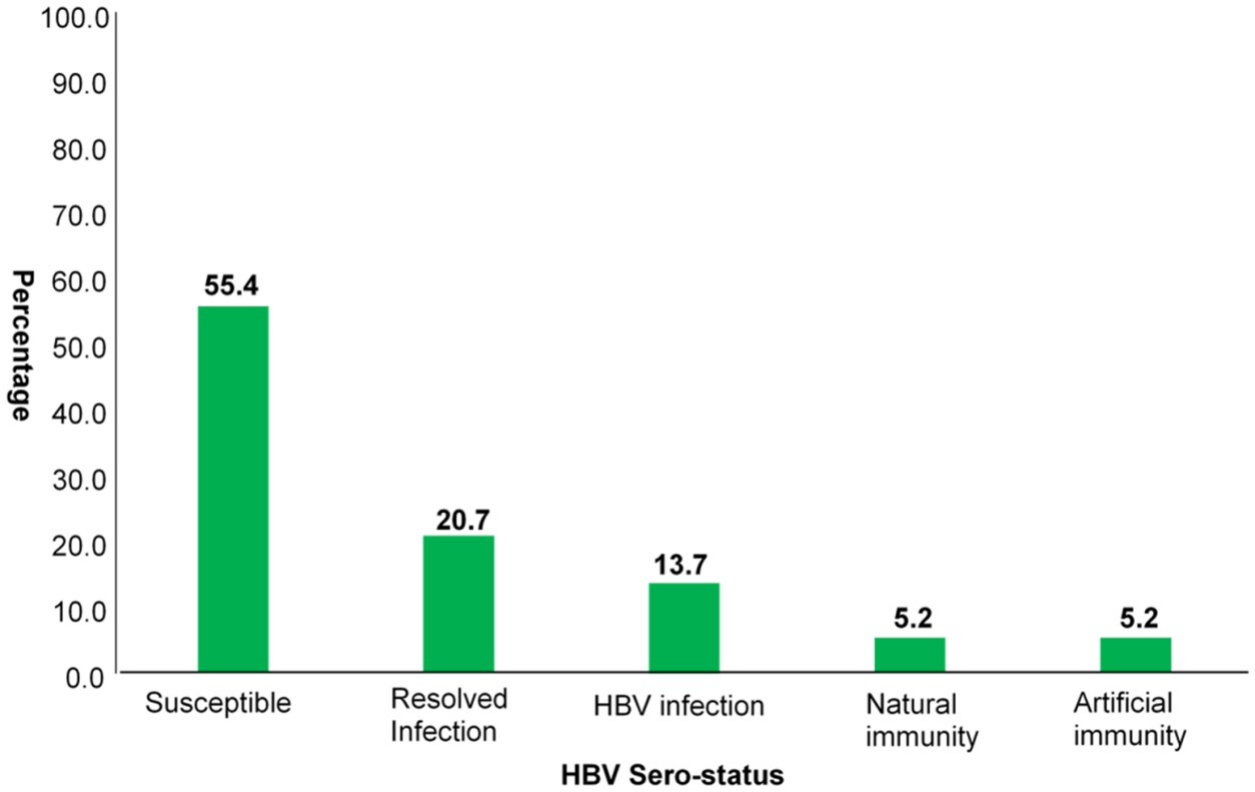
HBV sero-status of FCT inmates.

More than half of the respondents (55.4% (95% CI: 49.2–61.4)) were found to be susceptible to HBV infection, 13.7% (95% CI: 9.8–18.3) have HBV infection, while 10.3% (95% CI: 7.0–14.6) had acquired immunity either due to successful vaccination or previous infection. (Fig 2) HBV sero-status of FCT inmates and Table 2.

**Table 2:** Frequency of FCT inmates HBV markers result.

HBV Profile	HBsAg+	HBsAb+	HBeAg+	HBeAb+	HBcAb+	Frequency(%)
HBV infection	37	0	3	22	36	37(13.7)
Susceptible	0	0	0	0	0	150(55.4)
Past infection with natural immunity	0	14	0	1	13	14 (5.2)
Past infection without natural immunity	0	0	0	1	56	56(20.7)
Artificial immunity	0	14	0	0	0	14(5.2)
**Total**	**37**	**28**	**3**	**24**	**105**	**271**

Inmates within the age-group ≤ 25 years were 3 times (95% CI: 1.5–7.6) more likely to be infected than inmates ≥ 25 years. Likewise, married participants were 2 times (95% CI: 1.0–4.3) more likely to be infected than their unmarried counterpart. Furthermore, inmates with history of alcohol consumption were 3 times more likely to have HBV infections than inmates without history of alcohol consumption (95% CI: 1.1–6.2).

Although statistically insignificant, 5 (17.9%) and 5 (16.1%) participants who have ever had a blood-oath and body piercing respectively were found to have HBV infections likewise other behavioural risk factors such as history of illicit drug use, local shaving of hair, use of intravenous drugs and patronizing local manicure/pedicure vendors (Table 3).

**Table 3:** Association between HBV infection and socio-demographic/ behavioural risk factors.

Variables	Positive (%)	Negative (%)	OR (95% CI)
**Age**			**3.4 (1.5–7.6)**
≤25 years	11(29.7)	26 (70.3)	
≥25 years	26(11.1)	208 (88.9)	
**Religion**			1.2 (0.2–2.6)
Christianity	26 (14.4)	154 (85.6)	
Islam	11 (12.1)	80 (87.9)	
**Marital Status**			**2.1 (1.0–4.3)**
Ever married	25 (17.6)	117 (82.4)	
Never married	12 (9.3)	117 (90.7)	
**History of alcohol use**			**2.6 (1.1–6.2)**
Yes	30 (17.1)	145 (82.9)	
No	7 (7.3)	89 (92.7)	
**Ever had a blood oath**	5 (17.9)	23 (82.1)	0.7 (0.2–2.0)
Yes	32 (13.2)	211 (86.8)	
No			
**Patronize local manicure/pedicure vendors**			1.1 (0.5–2.2)
Yes	16 (13.2)	105 (86.8)	
No	21 (14.0)	129 (86.0)	
**Shave hair locally**			1.0 (0.4–1.9)
Yes	19 (13.9)	118 (86.1)	
No	18 (13.4)	116 (86.6)	
**Ever had body piercing**			0.8 (0.3–2.2)
Yes	5 (16.1)	26 (83.9)	
No	32 (13.3)	208 (86.7)	
**Ever had blood transfusion**			0.6 (0.1–2.6)
Yes	2 (8.7)	21 (91.3)	
No	35 (14.1)	213 (85.9)	
**Ever been tattooed**			1.5 (0.5–4.4)
Yes	4 (10.3)	35 (89.7)	
No	33 (14.2)	199 (85.8)	
**Intravenous drug use**			1.8 (0.2–14.2)
Yes	1 (7.7)	12 (92.3)	
No	36 (9.7)	222 (90.3)	
**History of illicit drug use**			1.2 (0.6–2.4)
Yes	20 (14.6)	117 (85.4)	
No	17 (12.7)	117 (87.3)	

Majority (n = 165, (60.9%)) participants reported congestion i.e. sharing of cell with ≥10 other inmates.

Association between HBV infection and sexual/prison related behavioural risk factors shows only (n = 1, (10%)) inmates who have been previously incarcerated have HBV infection. Equally, 4 (15.7%) inmates who share personal belonging with other inmates were found to have HBV infections while 5 (15.2%) inmates who share sharp objects like razor with other inmates had HBV infections though not statistically significant. None of the sexual risk factors were found to be significantly associated with HBV infection. However, 33 (16.1%) inmates who have multiple sexual partners and 10 (15.4%) inmates who had ever engaged in transactional sex had HBV infections. (Table 4).

**Table 4:** Association between HBV infection and sexual/prison related behavioural risk factors risk factors.

Variable	Positive (%)	Negative (%)	OR (95% CI)
**Previous incarceration**			0.7 (0.1–5.6)
Yes	1(10.0)	9 (90.0)	
No	36 (13.8)	225 (86.2)	
**Duration of incarceration**			1.0 (0.5–2.3)
<6 months	10 (13.2)	66 (86.6)	
>6months	27 (13.9)	168 (86.1)	
**Share personal belongings with other inmates**			0.8 (0.2–2.4)
Yes	4 (16.7)	20 (83.3)	
No	33 (13.4)	214 (86.6)	
**Share sharp objects with other inmates**			0.8 (0.4–1.7)
Yes	5 (15.2)	86 (84.8)	
No	22 (12.8)	152 (87.2)	
**Number of lifetime sexual partners**			0.4 (0.1–1.2)
0–1	4 (7.0)	64 (93.0)	
>1	33 (16.4)	170 (83.6)	
**Sex with non-marital partner**			0.6 (0.3–1.4)
Yes	10 (18.5)	44 (81.5)	
No	27 (12.4)	190 (85.1)	
**Condom use during extramarital sex**			0.9 (0.4–1.8)
Always	13 (14.9)	74 (85.1)	
Sometimes/No	24 (13.4)	160 (86.6)	
**Ever engaged in homosexuality**			*
Yes	0 (0.0)	4 (100)	
No	37 (13.9)	230 (86.1)	
**History of STI**			0.9 (0.4–2.4)
Yes	7 (13.0)	47 (87.0)	
No	30 (13.8)	187 (86.2)	
**Ever had transactional sex**			1.2 (0.5–2.6)
Yes	10 (15.4)	55 (84.6)	
No	27 (13.1)	179 (86.9)	

* Not calculable.

Multivariable adjusted logistic regression analysis was further used for exploring risk factors associated with HBV infection, younger inmates within age-group <25 years were 8 times as likely to have HBV infection than inmates >25years (aOR = 8.0, 95% CI: 2.9–22.3), Inmates with history of alcohol intake were 3 times as likely to have HBV infection than inmates who do not take alcohol (aOR = 3.4, 95% CI: 1.3–8.4). Inmates who have ever been married were 4 times as likely to have HBV infection as inmate who were never married (aOR = 4.2, 95% CI: 1.7–10.4). There was however no significant association (OR = 1.0, 95% CI: 0.9–1.0) between inmates who have multiple sex partners and HBV infection. (Table 5).

**Table 5:** Multivariate analysis of factors associated with HBV infection.

Variable	aOR	95% CI
**Age**		
≤25 years	8.0	**2.9–22.3**
≥25 years		
**History of alcohol use**		
Yes	3.4	**1.3–8.4**
No		
**Marital status**		
Ever Married	4.2	**1.7–10.4**
Never Married		
**Life time sexual partner**		
0–1	1.0	0.9–1.0
2 and above		

## Discussion

The HBV seroprevalence of 13.7% obtained in this study is slightly higher than the 12.2% HBV infection reported in the national survey among apparently healthy population in Nigeria [12]. Overcrowding, poor literacy rates, low socioeconomic status and poor health facilities in prisons remains a concern in developing countries, and is a key causative factor for a myriad of other problems which ultimately turn these custodial settings into fertile breeding grounds for infectious diseases such as Hepatitis B. Prison data provide definitive epidemiological evidence for this cohort of high risk population’s at lower cost than comparable community surveys [27–30].

The HBV current infection seroprevalence of 13.7% obtained in this study is consistent with similarly high seroprevalence of 17.4% in Ghana, 10.9% in Lomé and 14.1% Dakar reported among prison inmates [3, 21]. However, a higher seroprevalence of 23.0% was obtained among inmates in Nasarawa state, Nigeria [1]. On the contrary, lower HBV seroprevalence of 2.0%, 3.2%, 8.0%, 6.7% and 6.5% found in Georgia- US, Australia, United Kingdom, Italy and Greece respectively, could be due to low HBV prevalence in developed countries when compared to sub-Saharan Africa [4, 31–34].

The overall HBcAb seroprevalence of 38.4% is similar to the findings of 31% and 39.7% in Australia and Spain, however higher than the 17.5% found in a similar study in incarcerated populations in Minas Gerais State-Brazil, 8.7% in Ireland, an average 22.8% in United States of America, although relatively lower than the 57.6% in Greece and 52.7% in Italy [4, 31–39].

The high sero HBsAg and HBcAb prevalence found among FCT inmate’s prisoners compared to prisoners in the United States of America, Europe and Australia probably reflects the current absence of any harm reduction and HBV preventive interventions in Nigerian prisons when likened to variable extent of implementation of such interventions in prisons of developed world. Additionally, it could also be attributed largely to low HBV endemicity in these regions compared to high endemicity of HBV in the sub-Saharan region in variably accounting for the higher cases of liver cancers reported in sub-Saharan Africa [31, 33, 36].

Conversely, higher HBcAb of 57.6% and 52.7% found in Italy and Greece respectively may be attributed to a possible practice of IDU by inmates–a high-risk behaviour deficient among our study participants [31, 32].

Our findings reveal higher HBV current infection rates compared to 12.2% found among asymptomatic Nigerians in first Hepatitis national survey but consistent with the range of 9–39% documented in various studies done across Nigeria in different sub populations mainly healthy blood donors, pregnant women and other high risk group such as commercial sex workers, health care workers and injection drug users [12, 40–45]. This study supports previous reports that prisoners represent a high risk group for blood borne diseases like HBV. These findings are suggestive that horizontal transmission aided by cultural or behavioural factors is the main determinant of HBV prevalence in Nigeria [1, 46, 47].

Our study found that 119 (43.9%) inmates had one or more serologic markers of HBV infection, while 56 (20.7%) have been previously exposed. This in conformity with the already established high prevalence HBV infection among inmates and elucidates the report by that 87% of the Nigerian population have at least one HBV serologic marker by the age of 40 years [48].

The high HBV seroprevalence 37 (13.7%) in this study of which 3 (8.1%) are asymptomatic HBeAg seropositive which connotes high infectivity, implies potentially infectious unaware inmates fuelling the HBV chain of transmission in an overcrowded prison facility. HBsAg and HBeAg determination in sera can be of immense contribution in the management of HBV infection since HBeAg described the infectivity status of the patient as well as chronic HBV infection. Hence, 1.1% of study participants positive for HBeAg in this study have higher chances of developing liver disease leading to cirrhosis and even primary liver cancer if not treated [49].

Furthermore, 22 (59.5%) HBsAg positive inmates had positive HBeAb, a marker of recent resolving infection. However, differentiating between acute (HBcAb-IgM) and chronic (HBcAb-IgG) infection is needed to substantiate this fact. This therefore advocates for a larger panel of HBV markers to avoid misclassification of HBV infection and ascertain if HBV infections were acquired intra or extra prison taking into cognisance inmate’s duration of incarceration [9]. The 38.4% prevalence rate of HBcAb marker recorded in our study is higher than the 11.4% reported by in Benin, and but similar to 38.2% Benue in Nigeria [9, 50].

The high prevalence of HBV susceptibility (56.5%) among study participants raises the exigent need for HBV vaccination in prison as to protect this high risk vulnerable subset of inmates given the high rate of exposure of inmates to HBV risk factors before and during incarceration.

The 5.2% of natural HBsAb found among apparently healthy inmates indicates past HBV infection and protection against subsequent HBV re-infection [25]. Furthermore, the 5.2% artificial immunity found in our study though not truly reflective of the participant’s response on vaccination status could be attributed to recall bias, also explicates the efficacy of vaccination in prevention of the disease and thus draws attention to the gap in HBV vaccination coverage in FCT prison. The American Advisory Committee on immunization and the Centers for Disease Control and prevention strongly advise hepatitis B vaccinations for inmates of long-term correctional facilities and IDUs [51]. This study provides basis for effective and evidence-based interventions that will promote reduction in HBV infection related morbidity and mortality in Nigerian prisons. Such interventions include introduction and promotion of universal access to HBV screening, care and vaccination of all susceptible inmates [1, 3, 4].

This study showed statistical significance between Hepatitis B virus infection and age group. Younger inmates ≤25 years were 8 times more likely to have HBV infections than older inmates, moreso, incarcerated youths have a higher prevalence of behaviours that may predispose them at risk of HBV infections [1, 52].

Inmates with history of alcohol intake were 3 times more likely to have HBV infection than their counterparts. Alcohol inhibition causes impaired decision making leading to increased HBV risk related behaviours [53].

We also found that inmates who have ever been married were 4 times more likely to have HBV infection than inmate who were never married. Unprotected sex is a known of mode of HBV transmission and marriage provides a means of unprotected sex which could increase the chances of exposure and transmission of HBV [43].

Surprisingly, only 4.4% of our participants admitted to having ever injected drugs, going by reports that IDU is a common practice among inmates [33, 54]. This is consistent with the assertion by that IDU is infrequent in sub-Saharan Africa [55]. IDU has been implicated as major risk behaviour for HBV transmission among prison inmates. Although none of our subjects confirmed the practice of IDU intra prison, it is possible this probably happens among Nigerian prisoners but at a very minimal level, not enough to influence the result [1]. However, most of our respondents admitted to smoking of illicit drugs such marijuana (*wiwi)*.

Although no statistical significant association was found between inmates who have multiple sex partners and HBV infection, 75% of participants reported multiple sexual partners while 68% admitted inconsistent condom use during extramarital sex. Growing evidence also suggests that in general, prisoners are more sexually active in the community than the general population with a higher number of sexual partners and lower use of condoms [47, 39].

Surprisingly, only 1.5% of our subjects admitted to having ever involved in homosexuality although Kuje prison is a male only prison. However, because homosexuality is prohibited in correctional facilities as well as a criminal offence in Nigeria, such exposures were probably underreported by respondents. Also, underreporting might explain why these exposures were not strongly associated with HBV infection in this study. We also found that all inmates (14.5%) who have tattoos had acquired it prior to incarceration though prisoners are reported to frequently tattoo their skins out of boredom, and in the process share needles and ink. These high-risk behaviours place inmates at increased risk of infection with blood-borne viruses in comparison to the rest of the population [47].

It is estimated that Kuje prison accommodates about 3 times its originally designated capacity such that over 50% of our respondents share cells with ≤ 20 inmates and a quarter with ≤150 inmates. These poor living conditions prevailing in prisons includes overcrowding leads to sexual activities and poor hygiene practices such as sharing of unsterile razors and needles for tattooing, may contribute significantly to the transmission of HBV infection [56]. These risk factors are potentials for HBV transmission which exists in clustered prison settings. Clustering ultimately predisposes to events such as altercations which may have the propensity to result in exposures to HBV contaminated body fluids [4, 57, 58].

The limitations encountered in this study include response bias; similar to most behavioural surveys, the issue of under-reporting of prohibited risk behaviours such as illicit drug use, homosexuality abound due to victimization, socio-cultural and religious beliefs concern of the study participants (despite all assurances of confidentiality during the study). The inability to differentiate between inmates with current acute infection and those with chronically infected or an occult HBV infection was encountered as HBV genotyping data or HBV DNA quantification and HBcAb- IgG and HBcAb- IgM were not assayed. Owing to the cross-sectional study design used it was difficult to definitively ascertain if the prison inmates acquired the infection within or outside of the prison. Moreover, all participants were males, which limit the generalizability of the results to the female inmate population.

## Conclusion

This study found a high HBV sero-prevalence among inmates which indicates hyper-endemicity of HBV infection in FCT prison. More than half of the study participants were found to be susceptible while only about 10% had acquired either natural or artificial HBV immunity. Younger age-group (≤25 years), history of alcohol use, and ever been married were factors significantly associated with HBV infection.

The high HBV sero-status of Kuje inmates is a cause for concern despite the long standing strong recommendation by the American advisory committee on immunization and the US centers for disease control and prevention for hepatitis B vaccinations of long-term correctional facilities inmates [51]. Correspondingly, the international community has generally accepted that prisoners retain all their fundamental human rights and should not be marginalised due to incarceration. Thus, the need to introduce appropriate research guided effective prison-based HBV infection prevention strategies such as health education, screening, vaccination and care in addition to risk reduction programs and policies geared towards reducing prison populations in Nigerian is urgent and long overdue [59–61].

## Supporting information

S1 File Study questionnaire. (DOCX)

S2 File CDC algorithm for HBV infection serological patterns. (DOCX)

S3 File Study data. (XLSX)

## Abbreviations

AOR: Adjusted Odds Ratio
AWT: Awaiting Trial
CDC: Centers for Disease Control and Prevention
CI: Confidence Interval
ELISA: Enzyme Linked Immunosorbent Assay
FCT: Federal Capital Territory
HBcAb: Hepatitis B core Antibody
HBcAg: Hepatitis B core Antigen
HBeAb: Hepatitis B e Antibody
HBeAg: Hepatitis B e Antigen
HBsAg: Hepatitis B Surface Antigen
HBV: Hepatitis B Virus
HIV: Human Immunodeficiency Virus
IDU: Injection Drug Use/User
IgG: Immunoglobulin G
IgM: Immunoglobulin M
OR: Odds Ratio
PCR: Polymerase Chain Reaction
PPS: Probability Proportionate size
WHO: World Health Organization
