# Supplementary material for: Serological markers and risk factors associated with Hepatitis B virus infection among Federal Capital Territory prison inmates, Nigeria: Should we be concerned?
Source: PLoS One. 2021 Mar 11;16(3):e0248045. doi: 10.1371/journal.pone.0248045 (PMC7951833; doi:10.1371/journal.pone.0248045)
Supplement: S1 File — (DOCX) [file pone.0248045.s001.docx]

## Questionnaire

**Serological Markers and Factors associated with Hepatitis B Virus (HBV) infection amongst Kuje Prison Inmates Federal Capital Territory**

Date_________________ INMATE CODE: ________

Inmate status Awaiting trial Convict

Good day, we are conducting a survey on the prevalence of Hepatitis B virus among Kuje Prison inmates. You are not compelled to participate; however we will be grateful if you could kindly oblige us a few minutes of your time. All information supplied would be treated as strictly confidential.

**SECTION 1**

**DEMOGRAPHIC DATA**

1. Age (as at last birthday): ____________________
2. Sex: Male
3. Marital status
4. Single Married Separated Divorced Widowed Cohabiting

4b. If married how many wives _________________

5. State of origin __________________

6. Tribe: Hausa Igbo Yoruba Others specify _________________

7. Religion: Christian Muslim Others specify _______________

8. What was your occupation before imprisonment?

Public servant Self employed Retired Unemployed others specify ___________

9. If employed/self-employed, what do you do? ____________________________

10. What is your highest educational qualification?

No formal education Primary Secondary Tertiary Quaranic

**SECTION 2**

**Knowledge of Hepatitis B**

11. Have you heard about Hepatitis B Infection? Yes No

12. If Yes, what is your **MAIN** source of information about Hepatitis B?

School/Education Friends/colleagues Family member Television/Radio

Church/Mosque Books/Magazines Hospitals/Clinic Prison

13. Hepatitis B can be transmitted through?

Body contact Sexual intercourse Sharing of personal belongings Mother to child

Sharing of sharp objects Blood transfusion Don’t know

14. Hepatitis B can be prevented through?

Vaccination Use of condom Not sharing sharp objects/needles Limiting sex to a faithful uninfected partner By screening blood prior to transfusion Don’t know

15. Hepatitis B can be diagnosed by a blood test? Yes No Don’t know

16. Do you think that Hepatitis B is curable? Yes No Don’t know

**SECTION 3**

**RISK FACTORS**

11a. How long have you been imprisoned? ___________________

11b.Type of crime committed? ___________________________________

11c. Have you ever been imprisoned before? Yes No

12. How many inmates do you share your cell with? ___________

13. Do you have tribal marks/ scarification? Yes No

14. Have you ever had a blood oath? Yes No

15. Do you patronize local manicure/pedicure vendors? Yes No

16. Do you shave your hair locally? Yes No

17. Do you share personal belongings with other inmates e.g Tooth brush ,Towels?

Yes No

18. Do you share sharp objects e.g., Needles Razor blades clippers with your fellow inmates?

Yes No

19a. Do you have body piercing? Yes No

19b. If yes, did you acquire it in prison? Yes No

20a. Do you have Tattoo on your body?  Yes No

20b. If yes, did you get in prison? Yes No

21a.Do you inject intravenous drugs? Yes No

21b.If yes, do you share needles with partners? Yes No

22. How many sexual partners have you had in your lifetime? _________

23. Have you had sexual intercourse with someone who is not your spouse(s) or your regular partner within the last 6 months? Yes No Not sure

24. Do you use condom when having extramarital sex or with someone who isn’t your regular partner? Always No Sometimes

25. Your sexual partners are? Male only Female only Both

26. Have you ever enagaged in homosexuality (men sleeping with men) Yes No

27. Have been diagnosed with sexually transmitted infections (STIs) symptoms include painful urination and discharge from Penis?

Yes No Don’t know

28. Have you ever exchanged sex for money, drugs or a need? Yes No

29. Had sex while under the influence of illicit drugs or alcohol Yes No

29a.Do you take alcohol? Yes No

29b.Do you take illicit drugs like Weed, Igbo, and Cocaine? Yes No

30. Have you ever received blood transfusion? Yes No

31. Have you ever tested for HIV? Yes No

31a. If Yes what was your result Positive Negative

**SECTION 4**

**HBV TEST AND VACCINATION HISTORY**

31a.Have you ever been tested for Hepatitis B? Yes No

31b.If yes what was your result? Negative Positive

32a. Have you ever received hepatitis B vaccine?

Yes No Not Sure

32b. If yes, which year did you receive the vaccine (year)? ………………

32c. How many doses of the vaccine did you receive?

One dose Two doses Three doses More than 3 doses Not sure

**Result**

HBsAg HBsAb HBcAb HBeAg HBeAb __________

HBV Status ­­­­­­­­­­­­­­­­­­­­­­­­_______________________________
