# Supplementary material for: Serological markers and risk factors associated with Hepatitis B virus infection among Federal Capital Territory prison inmates, Nigeria: Should we be concerned?
Source: PLoS One. 2021 Mar 11;16(3):e0248045. doi: 10.1371/journal.pone.0248045 (PMC7951833; doi:10.1371/journal.pone.0248045)
Supplement: S2 File — (DOCX) [file pone.0248045.s002.docx]

HBV infection serological patterns and interpretations

| HBsAg | HBsAb | HBeAg | HBeAb | HBcAb | INTERPRETATION |
| --- | --- | --- | --- | --- | --- |
|  |  |  |  |  |  |
| - | - | - | - | - | Susceptible |
| + | - | +/- | _ | +(IgM\IgG) | Current infection (Acute/Chronic) |
| + | - | + | - | +(IgM/IgG) | Acute/Chronic infection (with active viral replication) |
| + | - | - | + | +(IgM/IgG) | Acute/Chronic infection (with low /no viral replication) |
| - | + | - | + / - | +(IgG) | Immunity due to resolved infection |
| - | + | - | - | - | Immunity due to vaccination |
| - | - | - | - | +(IgG) | Resolved/Past infection |

Source: CDC website: www.cdc.gov/hepatitis/HBV
